# Lnc-SNHG16/miR-128 axis modulates malignant phenotype through WNT/β-catenin pathway in cervical cancer cells

**DOI:** 10.7150/jca.40319

**Published:** 2020-02-03

**Authors:** Wu Wu, Li Guo, Zhenlong Liang, Yuanbin Liu, Zhi Yao

**Affiliations:** 1 Department of Immunology, Tianjin Key Laboratory of Cellular and Molecular Immunology, Key Laboratory of Educational Ministry of China, Tianjin Medical University, Tianjin 300051, China.; 2 Department of Clinical Laboratory, Laigang Hospital, Jinan 271103, China.; 3 Department of Clinical Laboratory, Chinese PLA General Hospital, Beijing 100853, China.; 4 Department of Clinical Laboratory, Qilu Hospital of Shandong University, Jinan 250002, China.

**Keywords:** lnc-SNHG16, miR-128, GSPT1, WNT3A, WNT/β-catenin, cervical cancer

## Abstract

**Background**: The lnc-SNHG16 serves as an oncogene and miR-128 acts as a tumor suppressor in various cancers. However, the functional role of lnc-SNHG16 and miR-128 in CC still remain unknown. This study aims to explore the expression level of lnc-SNHG16 and miR-128 and its biological roles in CC.

**Methods**: lnc-SNHG16, miR-128, GSPT1 and WNT3A expression were analyzed using quantitative real-time PCR and bioinformatics in cervical cancer tissues and cells. Cell Counting Kit-8, EdU staining, colony formation assay, western blot, Transwell, immunofluorescence, immunohistochemical staining, luciferase reporter assay, electrophoretic mobility shift, tumor xenograft, and flow cytometry assays were employed to investigate the mechanisms underlying the effect of Lnc-SNHG16/miR-128 axis on cervical cancer.

**Results**: lnc-SNHG16 was up-regulated in CC cell lines and tissues. lnc-SNHG16 knockdown inhibited proliferation, restrained the epithelial-mesenchymal transition (EMT) process by regulating cell apoptosis and cell cycle. The next study indicated that lnc-SNHG16 knockdown markedly increased miR-128 level which is down-regulated in CC. Moreover, miR-128 overexpression significantly inhibited proliferation, EMT process and tumor growth by directly targeting GSPT1 and WNT3A. Finally, lnc-SNHG16 activates but miR-128 inactivates the WNT/β-catenin pathways in CC cells.

**Conclusion**: Our data suggest that lnc-SNHG16/miR-128 axis modulates malignant phenotype of CC cells through WNT/β-catenin pathway.

## Introduction

Cervical cancer (CC) is the fourth most common gynecological malignancies worldwide, which also is the second most common type of cancer in females in developing countries 1, 2. Although the vaccine against HPV and treatment efficacy has approved and reduced the incidence of infection, CC mainly causes approximately 311,000 deaths every year worldwide 3. In previous years, the prompt operation, chemotherapy and radiotherapy have been elevated; however, due to the unclear molecular mechanisms of the genesis and development, CC maintains a public health concern all over the world.

Long non-coding RNAs (lncRNAs) are non-coding RNAs more than 200 nucleotides in length, play important roles in many biological functions, including epigenetic regulation, proliferation, cell apoptosis, autophagy, and so on 4-6. Lnc-SNHG16 is dysregulated in a variety of malignancies, including oral squamous cell carcinoma (OSCC) 7, osteosarcoma 8, non-small cell lung cancer (NSCLC) 9, bladder cancer 10, glioma 11, gastric cancer 12, pancreatic cancer 13, and so on 14. Recently, Chen et al has reported that LncRNA SNHG16 promotes hepatocellular carcinoma proliferation, migration and invasion by regulating miR-186 expression 15. However, Xu et al reported that lnc-SNHG16 overexpression inhibited cell proliferation and tumor growth *in vivo* and 5-FU chemoresistance in HCC 16. Recently, Zhu et al reported that lnc-SNHG16 promotes tumor progression, acts as an endogenous 'sponge' to regulate the miR-216A-5p/ZEB1 axis in CC 17.

In this study, lnc-SNHG16 was up-regulated in CC and lnc-SNHG16 knockdown inhibited cell proliferation, migration, and invasion and EMT process. Bioinformatics and molecular biological assays were used to investigate the relationship among lnc-SNHG16, miR-128, and its target genes of GSPT1 and WNT3A during the progress of tumorigenesis, and to illuminate the underlying molecular mechanisms. Taken together, we reported that lnc-SNHG16/miR-128 axis modulated the proliferation, migration and invasion and EMT process *in vitro* and tumor growth *in vivo* through the WNT/β-catenin pathway.

## Material and Methods

### Tissue Samples

Forty-eight pair of human cervical tissues were used in our study, which was approved by the ethical review committees of Tianjin Medical University (NO. 2017TMUH0191). The samples were received from Tianjin Medical University Cancer Institute and Hospital from December 2017 to December 2018. The written informed consents were obtained from all enrolled patients. Patients did not receive chemotherapy or radiotherapy before surgery and CC was confirmed by pathological examinations. Tissues were placed in the RNA preserving solution, overnight at 4°C, and preserved at -80 °C until RNA extraction. This study was approved by the Ethics Committee of Tianjin Medical University and conformed to the protocols in the Helsinki declaration.

### Cell culture and transfection

Normal human endocervical epithelial cell lines (Endl/E6E7) was obtained from Shanghai Medical College, Fudan University. Other cervical cancer cells used in this study were obtained from the American Type Culture Collection (ATCC). Endl/E6E7 was cultured in KER-SFM medium at 37°C in 5% CO2 supplemented with 10% FBS (Gibco). CC cells were cultured in RPMI 1640 (Invitrogen, Carlsbad, CA) supplemented with 10% FBS (Gibco), 100 μg/ml streptomycin, 100 U/ml penicillin in 5% CO2 at 37°C. Transfection assay was preceded using Lipofectamine^TM^ 2000 reagent (Invitrogen) according to the manufacturer's protocol.

### RT-qPCR assay

Total RNAs from tissues and tissues were extracted with TRIzol (Sigma) reagent according to the protocols provided by corporation. Reversely transcription and qPCR were performed using miScript II RT kit (TaKaRa Biotechnology Co., Ltd., Dalian, China) and SYBR Premix Ex TaqTM II (Takara, Japan) on the ABI PRISM 7500 real-time PCR System. The primer sequences used were showed in Table 1.

### CCK8 assay

C33A and HeLa cells in the logarithmic growth phase were seeded into 96-well plate at 5000 cells per well one day prior to transfection. Then, 10 μl CCK-8 reagent (Sigma) was added into each well and incubated at 37°C for 4 h. The absorbance was detected to evaluate the relative cell viability at 450 nm using the microplate autoreader (Hitachi, Ltd., Tokyo, Japan).

### Transwell assays

C33A and HeLa cells were collected and suspended in serum-free medium after transfected with the indicated plasmids. At 24 h, the ability of migration was captured with the DM2500 bright field microscope (Olympus Corporation, Tokyo, Japan). For invasion, cells were seeded into the upper chamber of each transwell insert pre-coated with Matrigel (BD Biosciences). At 36 h, the ability of invasion was captured with the DM2500 bright field microscope (Olympus Corporation, Tokyo, Japan).

### Apoptosis and cell cycle assay

Cell apoptosis was detected by Annexin-V-FITC (fluorescein isothiocyanate) apoptosis detection kit (BD, Franklin Lakes, NJ, USA). Cell cycle was detected by Cell Cycle Detection Kit (Beckman Coulter, Brea, CA).

### *In vivo* tumorigenicity assay

Animal protocols were approved by Tianjin Medical University Animal Care and Use Committee (LLSP2017-0198). The methods were conducted in accordance with the approved guidelines. For the* in vivo* study, 6-week-old female BALB/c athymic nude mice (Institute of Zoology, Chinese Academy of Sciences, Shanghai, China) were used [n=30; divided into 5 groups (pcDNA3, miR-128, pSilencer, shR-GSPT1 and shR-WNT3A), 6 mice per group; weight, 20-30 g; maintenance conditions: Temperature, 18-29˚C; relative humidity, 50-60%; free access to clean food and water; and lighting for 10 h (lights turned on at 8:00 every day and turned off at 18:00)]. A total of 100 µl of miR-128 or its control cell suspension (3×10^6^ cell), shR-GSPT1 and shR-WNT3A or its control cell suspension (3×10^6^ cell) was subcutaneously inoculated into the ventral forearm of nude BALB/c mice. At 30 day, the weight of all tumors was detected. Tumor weight was measured using an electronic scale, and the Student's t-test was used to compare tumor growth among groups.

### Western blot assay

RIPA protein extraction reagent (Beyotime, Shanghai, China) with PMSF (Roche, Basel, Switzerland) was used to lysed HeLa cells. 10% SDS-PAGE was used to separate the protein and then transferred onto PVDF membranes (Millipore, Billerica, MA, USA). 5% non-fat milk was used to block the membranes for 2 h. The membranes were probed with anti-GSPT1 (Abcam, ab126090), anti-WNT3A (Abcam, ab28472), anti-ICAM1 (Abcam, ab222736), anti-Vimentin (Abcam, ab8069), anti-E-cadherin (Abcam, ab53013), anti-p-β-catenin (Abcam, ab11350), β-catenin (Abcam,ab32572), anti-c-myc (Abcam, ab39688), anti-cyclin D1 (Abcam, ab226977), anti-GAPDH (Abcam, ab181602) antibodies overnight at 4˚C. Subsequently, membranes were incubated with a HRP-conjugated secondary antibody (CST, #7074) for 1 h at 37˚C. Finally, the blots were visualized by ECL (Thermo Fisher Scientific) and detected using a ChemiDoc XRS imaging system. Band densities were analyzed using Image J software (National Institute of Health, Bethesda, MD, USA). Relative protein levels were determined by normalizing the densitometry value of the proteins of interest to that of GAPDH.

### EdU assay

EdU staining was performed using the Click-iTEdU imaging kit (Invitrogen) according to the manufacturer's instructions. Briefly, cells were exposed to 50 μM EdU for 2 h, then fixed with 4% formaldehyde. The cells were then treated with 2 mg/ mL glycine to neutralize the formaldehyde, then permeabilized with 0.5% Triton X-100. Finally, the cells were reacted with 100 μL of 1X Apollo reaction cocktail for 30 min, followed by incubation with 100 μL of Hoechst 33342 (5 μg/mL). Images were acquired using an Olympus IX-71 inverted microscope (Tokyo, Japan). The percentage of EdU-positive cells was calculated by dividing the number of EdU-positive cells by the number of Hoechst-stained cells.

### Fluorescent reporter assay

HeLa and C33A Cells were seeded in 24-well plates the day before transfection. The cells were first transfected with Anti-miR-128 or pri-miR-128 expression vectors and were then transfected with the lnc-SNHG16 EGFP reporter vector wild type (wt) or EGFP reporter vector mutant (mut) on the next day. The red fluorescence protein (RFP) expression vector pDsRed2-N1 (Clontech) was spiked in and used for normalization. The cells were lysed with immunoprecipitation assay lysis buffer 48 h after transfection, and the proteins were harvested. The intensity of EGFP /RFP fluorescence was detected with a Fluorescence Spectrophotometer F-4500 (HITACHI, Tokyo, Japan).

### Kaplan-Meier analysis

The Cancer Genome Atlas database (TCGA, https://cancergenome.nih.gov/), which is a publicly accessible online database. Kaplan-Meier analysis was used to the online software of Kaplan-Meier Plotter (http://kmplot.com/analysis/).

### Statistical analysis

The statistical analyses were practiced by GraphPad Prism (Version 5.0). All data are presented as the mean±standard deviation, and the experiments were repeated three times independently. Differences were analyzed with Student's t-test between two paired groups. For comparisons of three or more groups, one-way analysis of variance was followed by the Bonferroni post-hoc test for comparison of two selected treatment groups; the Dunnett's post-hoc test was used for comparisons of the other treatment groups with the corresponding controls. Associations between miR-128 or lnc-SNHG16 expression and clinicopathological characteristics were assessed using the χ2 test. A p value less than 0.05 was considered significant.

## Results

### lnc-SNHG16 was up-regulated in cervical cancer

**Figure 1:**
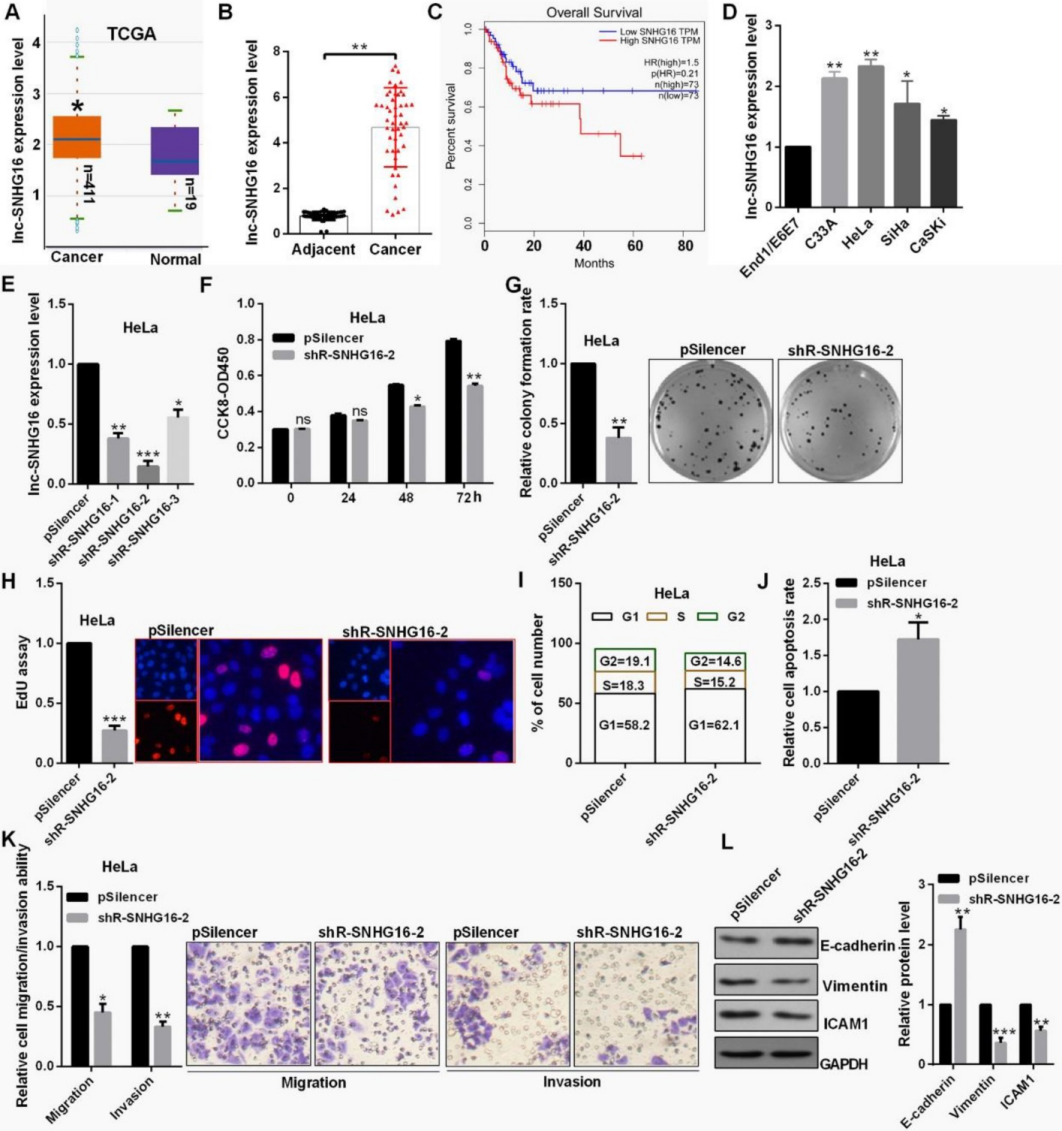
** lnc-SNHG16 functions an oncogene in cervical cancer.** (A) TCGA database showed the level of lnc-SNHG16 in CC. (B) RT-qPCR showed the lnc-SNHG16 level in tumor and adjacent tissues. (C) Kaplan Meier plotter software showed the overall survival of CC patients with high or low expression of lnc-SNHG16. (D) RT-qPCR showed the level of lnc-SNHG16 in CC cells. (E) Efficiency of shRNAs for lnc-SNHG16 was identified by RT-qPCR. (F) Effect of shR-SNHG16-2 on cell viabilities was determined in HeLa by CCK8. (G) Relative colony formation rates of cells transfected with shR-SNHG16-2 in HeLa were detected. (H) EdU staining showed the proliferation ability of HeLa cells transfected with shR-SNHG16-2. (I) Flow cytometric analysis showed that shR-SNHG16-2 results in the cell cycle blocking in HeLa cell. (J) Flow cytometric assay revealed that shR-SNHG16-2 increased the apoptosis of HeLa cells. (K) Transwell assays revealed that shR-SNHG16-2 suppressed cell invasion and migration ability. (L) Western blot analysis of the indicated protein expression levels following transfection with shR-SNHG16-2 in HeLa cells. Experiments were performed 3 times, and data are presented as means ± SD.*P<0.05; **P<0.01; ***P<0.001; ns, not significant.

lnc-SNHG16 was up-regulated in 411 CC patient tissues through ATCC database and 48 CC tissues by RT-qPCR (Fig. 1A and 1B). lnc-SNHG16 is significantly positively associated with TNM stage, tumor size, distant metastasis and the prognosis of survival in patients with CC (Fig. 1C, Table 2). lnc-SNHG16 was up-regulated in 4 CC cell lines (Fig. 1D).

### lnc-SNHG16 promoted cell proliferation and EMT process in cervical cancer cells

The level of lnc-SNHG16 was obvious reduced transfected with the shR-SNHG16-2 (Fig. 1E). shR-SNHG16-2 inhibited cell viability, decreased the colony formation ability and the positive EdU staining in HeLa cells (Fig. 1F, 1G and 1H). shR-SNHG16-2 transfection caused a decrease in cells at the S and G2 phase and an increase in cells at the G0/G1 phase in HeLa cells (Fig. 1I). lnc-SNHG16 downregulation markedly promoted cell apoptosis in HeLa cells (Fig. 1J). shR-SNHG16-2 transfection attenuated the migratory and invasive abilities of HeLa cells (Fig. 1K). Furthermore, when lnc-SNHG16 was inhibited transfected with shR-SNHG16-2, the mesenchymal marker vimentin exhibited a decreased expression in HeLa cells, while the epithelial marker E-cadherin exhibited an increased expression (Fig. 1L).

### lnc-SNHG16 could bind to miR-128

**Figure 2:**
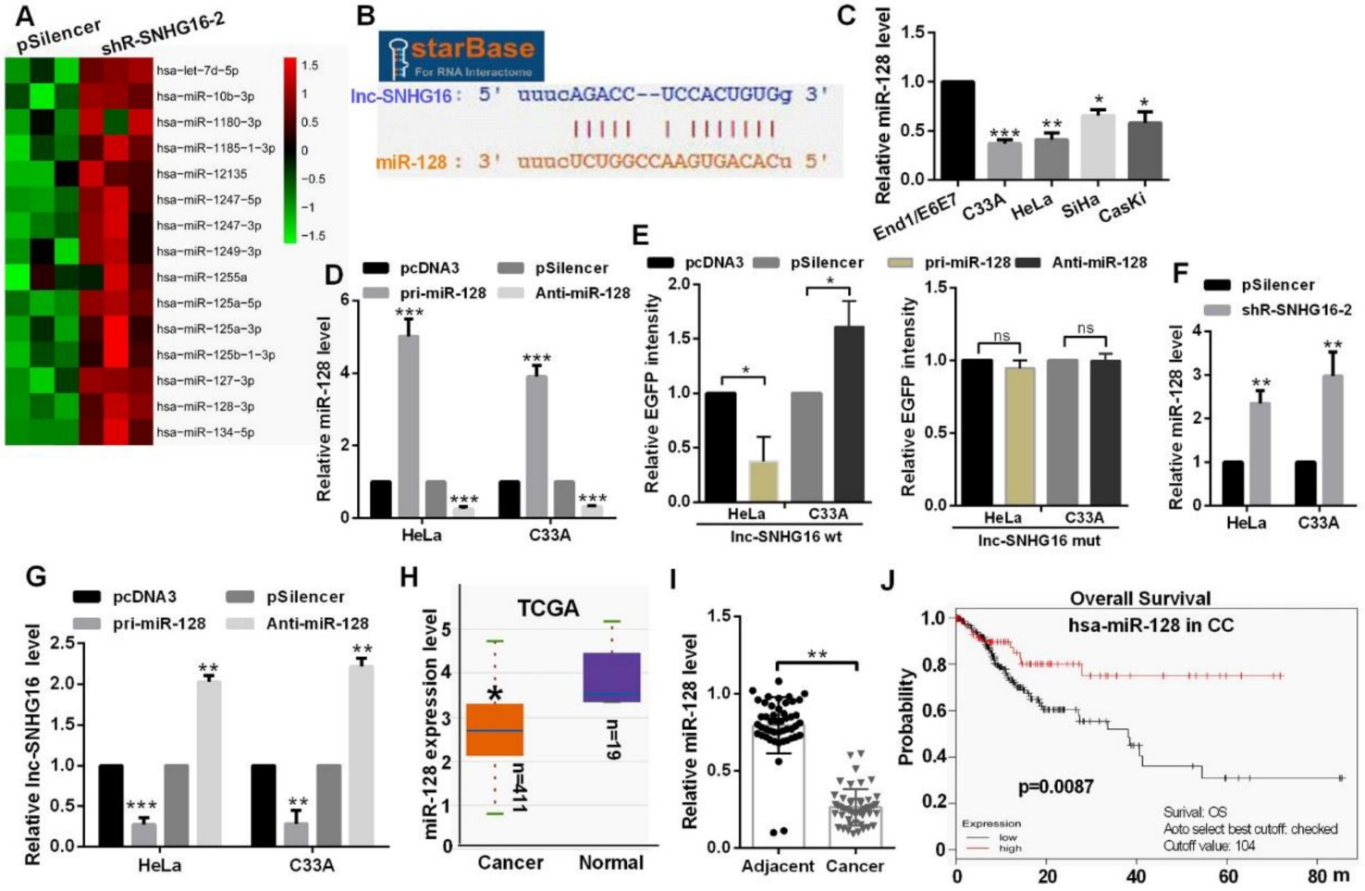
** lnc-SNHG16 could bind to miR-128.** (A) Hierarchical clustering showed the miRNA array profile in HeLa cells transfected with shR-SNHG16-2. (B) The relationship of miR-128 and lnc-SNHG16 was shown using StarBase. (C) RT-qPCR showed the miR-128 level in CC cells. (D) RT-qPCR showed the efficiency of pri-miR-128 or Anti-miR-128. (E) Fluorescence intensity was measured in CC cells co-transfected with pri-miR-128 or Anti-miR-128 and SNHG16 wt or mutant construct. (F) RT-qPCR assay showed the miR-128 level treated with shR-lnc-SNGH16-2. (G) RT-qPCR assay showed the lnc-SNHG16 mRNA level transfected with miR-128 overexpression or knockdown. (H) TCGA database showed the miR-128 level in CC patients and the control groups. (I) RT-qPCR showed the level of miR-128 in tumor tissues and the control groups. (J) Kaplan Meier plotter software showed the overall survival of CC patients with high or low expression of miR-128. Experiments were performed 3 times, and data are presented as means ± SD.*P<0.05; **P<0.01; ***P<0.001; ns, not significant.

Hierarchical clustering showed the top 15 up-regulated miRNAs in the shR-SNHG16-2 transfected group (Fig. 2A). StarBase database showed that lnc-SNHG16 carries putative miR-128 targeting sites (Fig. 2B). miR-128 was distinctly down-regulated in CC cells (Fig. 2C). Transfection of pri-miR-128 up-regulated miR-128 level and Anti-miR-128 transfection reduced the level of miR-128 in HeLa and C33A cells (Fig. 2D). In addition, we constructed EGFP reporters containing wild-type lnc-SNHG16 or lnc-SNHG16-mut. We found that transfection of pri-miR-128 reduced the EGFP activities of the wild-type lnc-SNHG16 reporter vector, but not empty vector or mutant reporter vector in HeLa and C33A cells (Fig. 2E). Knockdown of lnc-SNHG16 significantly increased the miR-128 expression level in HeLa and C33A cells (Fig. 2F). Then, miR-128 overexpression down-regulated the mRNA level of lnc-SNHG16, miR-128 knockdown has an opposite tendency in HeLa and C33A cells (Fig. 2G). miR-128 was down-regulated in 411 CC patients by TCGA and in CC tissues, which is significantly negatively associated with TNM stage, tumor size, and distant metastasis in patients with CC (Fig. 2H, 2I and Table 3). Kaplan Meier plotter software showed that the patients of CC with high miR-128 level had a favourable prognosis (Fig. 2J).

### miR-128 inhibited proliferation, EMT process and tumor growth

**Figure 3:**
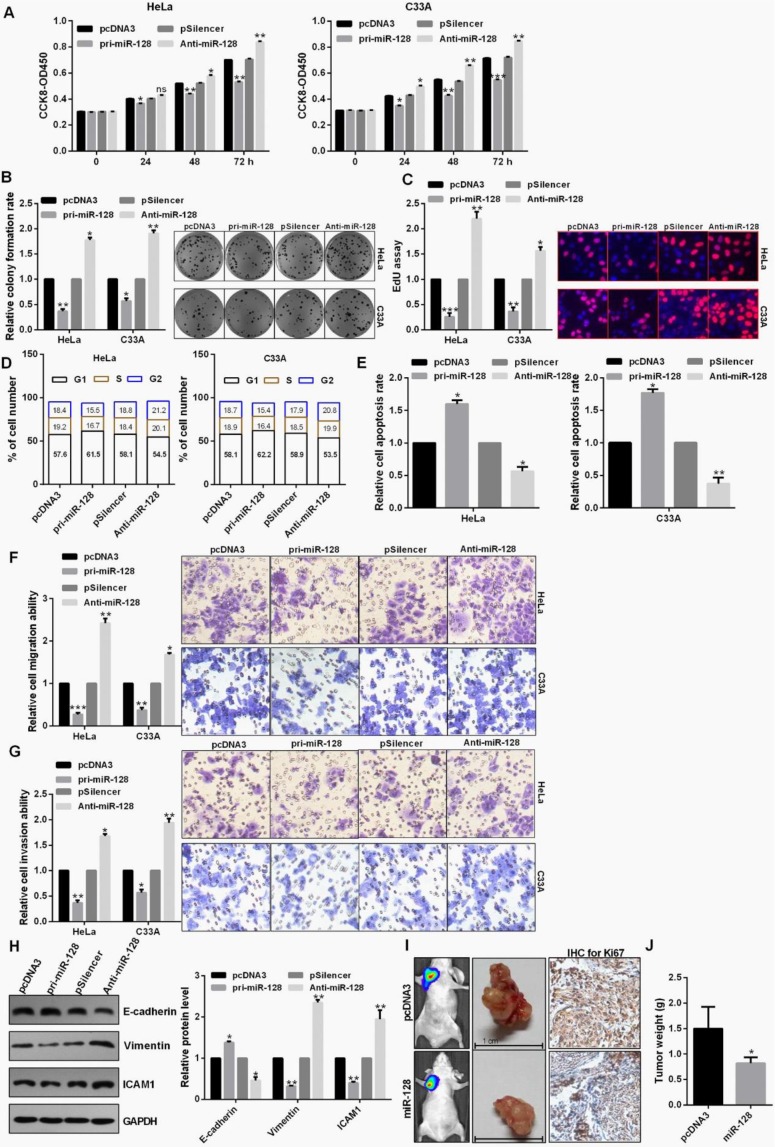
** miR-128 inhibited proliferation, EMT process and tumor growth.** (A) Effect of miR-128 on cellular viabilities of C33A and HeLa were detected by CCK8. (B and C) The proliferation ability of C33A and HeLa cells transfected with pri-miR-128 or Anti-miR-128 were detected by colony formation and EdU assay. (D) Flow cytometric analysis showed that overexpression of miR-128 leads to an increase of the G0/G1 phase. (E) Flow cytometric assay showed overexpression of miR-128 promoted cell apoptosis in CC cells. (F and G) Transwell assays revealed that miR-128 suppressed invasion and migration ability in HeLa and C33A cells. (H) Western blot analysis of the indicated protein expression levels in HeLa cells following treatment with pri-miR-128 or Anti-miR-128 and the control groups. (I and J) miR-128 overexpression inhibited tumor growth rate and tumor weight following with the down-regulation of Ki67 using IHC. Experiments were performed 3 times, and data are presented as means ± SD.*P<0.05; **P<0.01; ***P<0.001; ns, not significant.

miR-128 overexpression leads to cell growth inhibition in HeLa and C33A cells using CCK8 assay (Fig. 3A). Cell proliferation through the colony formation and EdU assay also exhibited an obvious attenuation of cell growth treated with pri-miR-128 in C33A and HeLa cells (Fig. 3B and 3C). Up-regulation of miR-128 could induce a significant G1 to S phase arrest in C33A and HeLa cells (Fig. 3D). In addition, apoptotic rate was increased in cells transfected with pri-miR-128 (Fig. 3E). Transwell assays demonstrated that miR-128 overexpression inhibited cell migration, invasion in C33A and HeLa cells (Fig. 3F and 3G). Pri-miR-128 decreased the protein expression level of Vimentin and ICAM1 and promoted the expression level of E-cadherin protein (Fig. 3H). The average weights of tumors in miR-128-transfected group were smaller than those in the control group and IHC revealed the significantly decreased Ki67 expression in miR-128-transfected group (Fig. 3I and 3J).

### miR-128 could directly target GSPT1 and WNT3A

**Figure 4:**
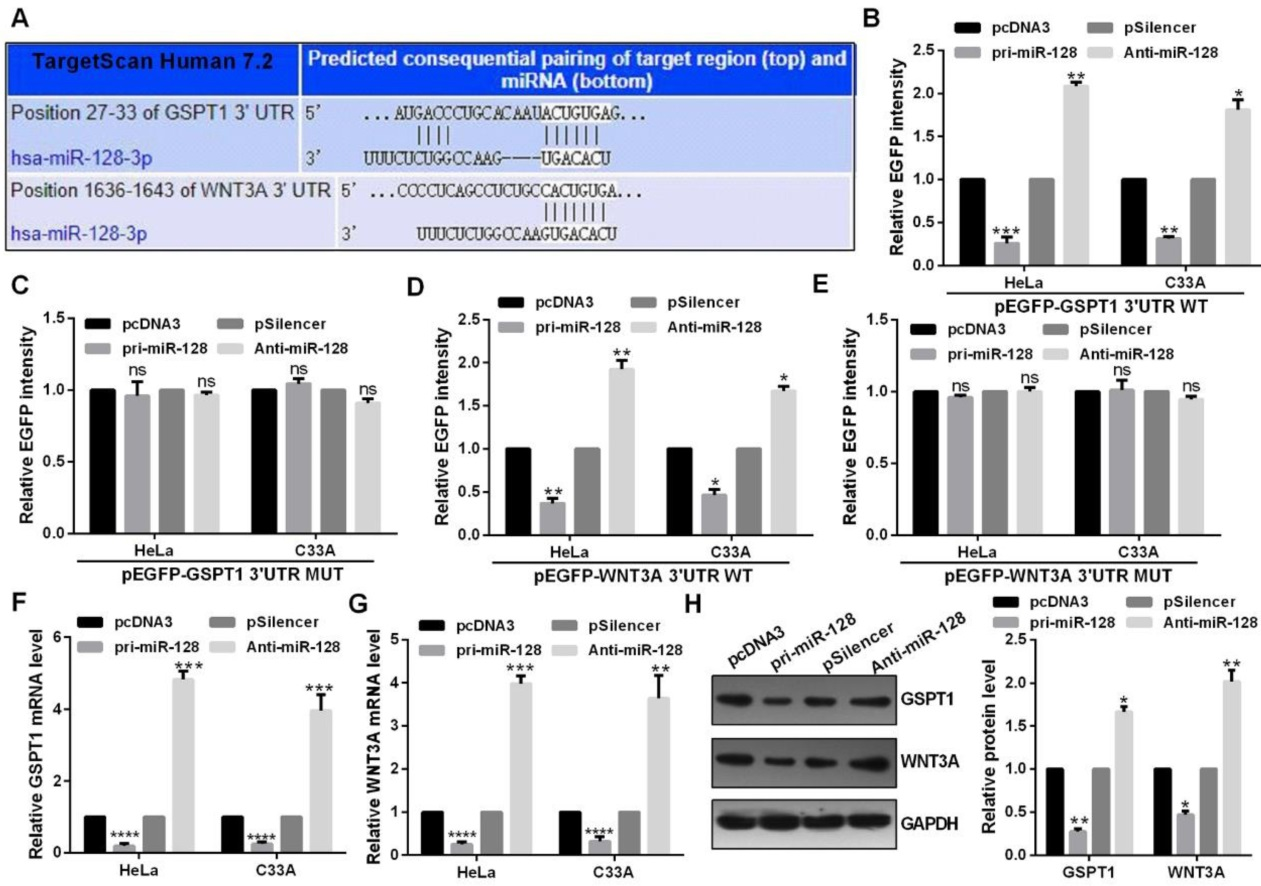
** miR-128 directly targeted GSPT1 and WNT3A.** (A) Predicted miR-128 binding sites in GSPT1 and WNT3A mRNA showed through TargetScan. (B and C) EGFP intensity of C33A and HeLa cells co-transfected with pri-miR-128 or Anti-miR-128 and the wild type or mutated 3'UTR of GSPT1. (D and E) EGFP intensity of C33A and HeLa cells co-transfected with pri-miR-128 or Anti-miR-128 and the wild type or mutated 3'UTR of WNT3A. (F) GSPT1 mRNA expression levels with indicated transfection were measured by RT-qPCR in C33A and HeLa cells. (G) WNT3A mRNA expression levels with indicated transfection were measured by RT-qPCR in C33A and HeLa cells. (H) GSPT1 and WNT3A protein level transfected with pri-miR-128 or Anti-miR-128 and respective controls were determined by western blot in C33A and HeLa cells. Experiments were performed 3 times, and data are presented as means ± SD.*P<0.05; **P<0.01; ***P<0.001; ns, not significant.

Highly conserved predicted binding sites were showed in the 3′UTRs of GSPT1 and WNT3A (Fig. 4A). miR-128 overexpression reduced the EGFP activity of the wide type miR-128 binding sites and failed to affect that of the plasmid carrying GSPT1 and WNT3A 3′UTRs mut in C33A and HeLa cells (Fig. 4B, 4C, 4D and 4E). Restoration of miR-128 expression suppressed the mRNA and protein expression of GSPT1 and WNT3A in C33A and HeLa cells (Fig. 4F, 4G and 4H).

### The level of GSPT1 and WNT3A in CC

**Figure 5:**
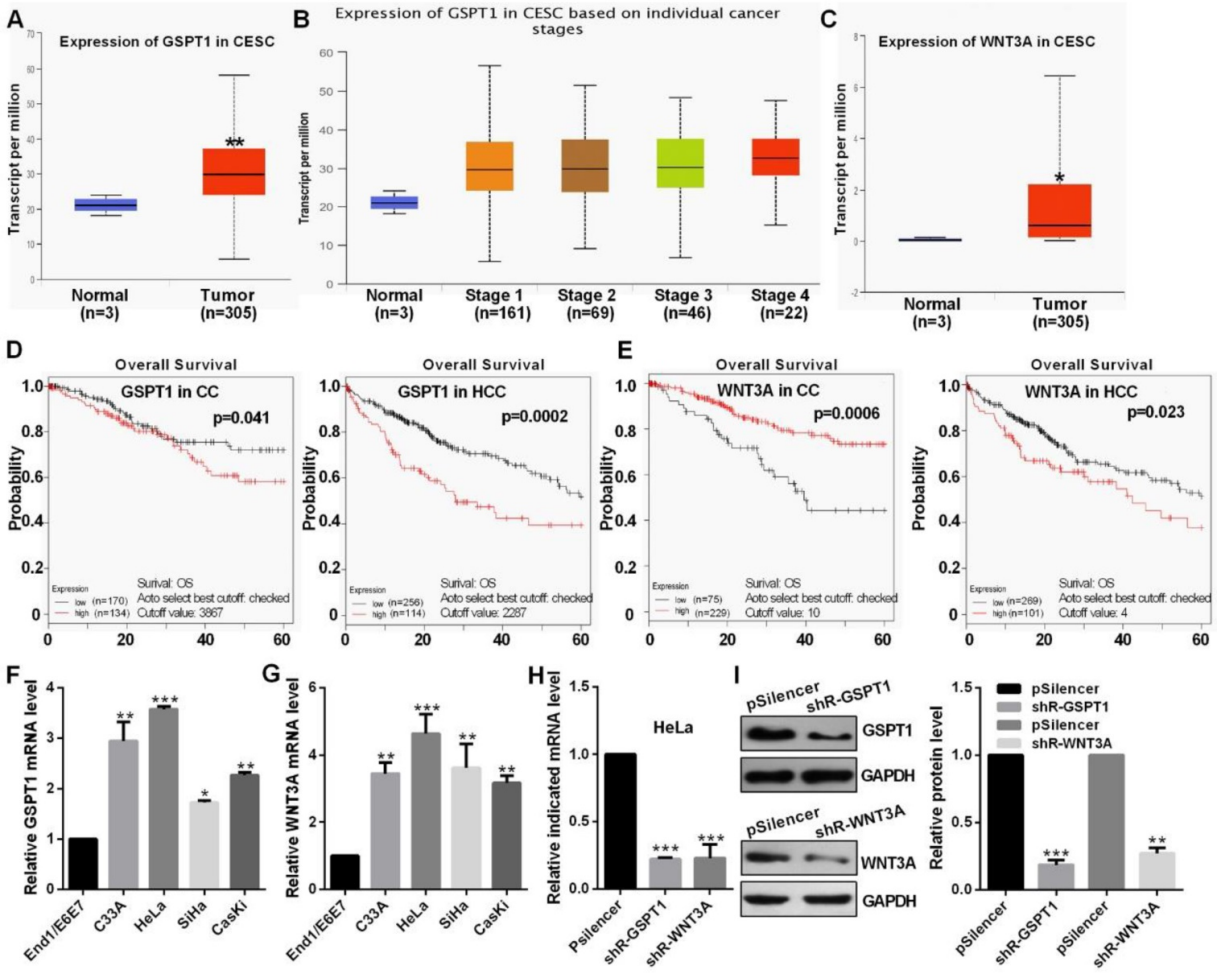
** The level of GSPT1 and WNT3A in CC.** (A and B) TCGA database showed the level of GSPT1 in CC. (C) TCGA database showed the level of WNT3A in CC. (D and E) Kaplan Meier plotter software showed the overall survival of patients with high or low expression of GSPT1 or WNT3A. (F and G) GSPT1 or WNT3A mRNA expression levels in CC cells were measured by RT-qPCR. (H) Efficiency of shR-GSPT1 and shR-WNT3A were identified by RT-qPCR. (I) Efficiency of shR-GSPT1 and shR-WNT3A were identified by western blot. Experiments were performed 3 times, and data are presented as means ± SD.*P<0.05; **P<0.01; ***P<0.001; ns, not significant.

TCGA datasets showed that GSPT1 and WNT3A expression were up-regulated and was positively related to the pathological stage (Fig. 5A, 5B and 5C). Kaplan Meier plotter software showed that the patients with high GSPT1 level had a poor prognosis in CC and HCC, the patients with low WNT3A level had a poor prognosis in CC but high WNT3A level had a poor prognosis HCC, which this argument is not clear (Fig. 5D and 5E). The expression levels of GSPT1 and WNT3A are up-regulated in CC cells (Fig. 5F and 5G). These shRNAs could silence the expression level of GSPT1 and WNT3A at mRNA and protein levels (Fig. 5H and 5I).

### The role of GSPT1 and WNT3A and the regulation of lnc-SNHG16 and miR-128 on WNT pathway

**Figure 6:**
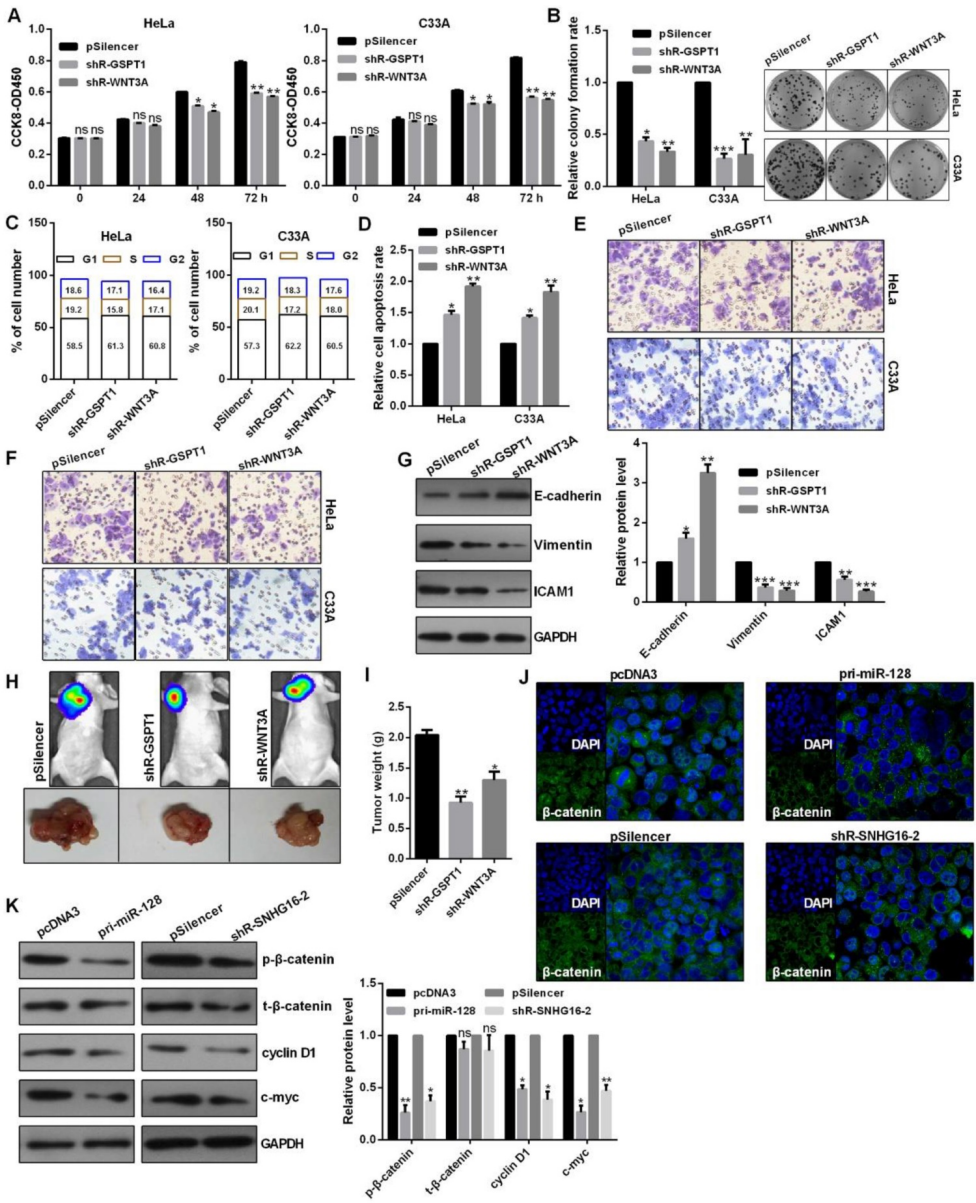
** The role of GSPT1 and WNT3A and the regulation of lnc-SNHG16 and miR-128 on WNT pathway.** (A) Effect of shR-GSPT1 and shR-WNT3A on cell viabilities was determined by CCK8 in C33A and HeLa. (B) Relative colony formation rates of C33A and HeLa cells transfected with shR-GSPT1 and shR-WNT3A were determined. (C) Flow cytometric analysis showed that shR-GSPT1 and shR-WNT3A lead to an increase in G0/G1 phase of C33A and HeLa cells. (D) Flow cytometric assay showed that shR-GSPT1 and shR-WNT3A significantly promoted the apoptosis rate of C33A and HeLa cells. (E and F) Transwell assays revealed that shR-GSPT1 and shR-WNT3A suppressed the invasion and migration ability. (G) Western blot analysis of the indicated protein expression levels following transfection with shR-GSPT1 and shR-WNT3A in HeLa cells. (H and I) Knockdown of GSPT1 and WNT3A inhibited tumor growth rate and tumor weight. (J) IF assay showed the distribution of β-catenin in HeLa cells transfected with the indicated plasmids. (K) Western blot showed the protein levels transfected with the indicated plasmids in HeLa cells. Experiments were performed 3 times, and data are presented as means ± SD.*P<0.05; **P<0.01; ***P<0.001; ns, not significant.

Knockdown of GSPT1 and WNT3A significantly inhibited the cell viability, colony formation ability, caused a cell cycle blocking (Fig. 6A, 6B and 6C). Knockdown of GSPT1 and WNT3A promoted cell apoptosis in C33A and HeLa cells (Fig. 6D). Knockdown of GSPT1 and WNT3A reduced the migratory and invasive abilities, increased E-cadherin expression, decreased vimentin expression in HeLa cells (Fig. 6E, 6F and 6G). The average weights of tumors were significantly smaller than those of control groups derived from the shRNAs-transfected HeLa cells (Fig. 6H and 6I). The distribution of β-catenin in nucleus was reduced in pri-miR-128-treated and shR-SNHG16-2-treated HeLa cells (Fig. 6J). Western blot revealed that the phosphorylated β-catenin (p-β-catenin), cyclin D1, c-myc protein were significantly inhibited by pri-miR-128 transfection and lnc-SNHG16 knockdown (Fig. 6K).

## Discussion

Increasing evidence have demonstrated that lncRNAs, which act as a competing endogenous RNA (ceRNA), could regulate the role of miRNAs in many cancers 18-20. For example, LINC00958 deletion prevented tumor initiation by sponging the miRNA-330-5p to regulate PAX8 expression in pancreatic cancer 21. In addition, lncRNA CACS15 by positively regulating ABCC1 contributed to oxaliplatin resistance through the miR-145 in CRC 22. Dong et al demonstrated that TINCR promoted trastuzumab resistance and EMT process by sponging the miRNA-125b in breast cancer 23. In this study, we demonstrated that lnc-SNHG16, which acts as a competing endogenous RNA of miR-128, regulates the CC progression. So, the precious results and our present study demonstrated lnc-SNHG16 could be target for prevention and therapeutics for CC in future.

miR-128 was low expression in osteosarcoma 24, glioma 25, ovarian cancer 26, thyroid cancer 27, HCC 28, CRC 29, gastric carcinoma (GC) 30. However, the role and mechanism of miR-128 remains unclear in CC. In this study, miR-128 was down-regulated in CC, which was negatively correlated with TNM stage, metastasis, tumor size, and poor prognosis. Overexpression of miR-128 inhibited tumor growth and cell proliferation through the regulation of apoptosis and cell cycle. Transwell analysis revealed that miR-128 overexpression inhibited cell invasion and migration. In addition, miR-128 could directly target GSPT1 and WNT3A in CC cells.

GSPT1 expression level was markedly increased in gastric cancer 31, colorectal cancer 32, and so on. Lee et al also reported that GSPT1 induced ASK1 (apoptosis signal-regulating kinase 1) activation 33. In this study, we reported that GSPT1, which was up-regulated in CC, facilitated cell proliferation, EMT and cell cycle process, tumor growth, inhibited the apoptosis of C33A and C33A cells.

WNT3A, one of the Wnt family members, plays important roles in regulating cellular functions of proliferation, apoptosis, cell cycle, and so on 34. WNT3A was up-regulated in GC 35, prostate cancer cells 36, breast cancer 37, 38, which was also up-regulated in oral squamous cell carcinoma and glioma stem cells compared with control nonmalignant tissues 39, 40. In this study, we reported that up-regulated WN3A functions as an oncogene in CC. Furthermore, we found that lnc-SNHG16 activated and miR-128 inactivated the WNT/β-catenin pathway by regulating the WNT3A.

In conclusion, we illuminated a new pathway in lnc-SNHG16 regulated miR-128 directly targeting GSPT1 and WNT3A on growth and metastasis through the WNT/β-catenin signaling pathway. Our experimental data also suggested that the lnc-SNHG16/miR‐128/WNT/β-catenin axis may be a promising therapeutic target for CC or other cancers.

## Figures and Tables

**Table 1:** Primers for this study

RT-qPCR primers	Primer Sequence (5'-3')
Lnc-SNHG16-Forward	CAGAATGCCATGGTTTCCCC
Lnc-SNHG16-Reverse	TGGCAAGAGACTTCCTGAGG
miR-128-RT	GTCGTATCCAGTGCAGGGTCCGAGGTGCACTGGATACGACAAAGAGA
miR-128-qPCR-Forward	TCACAGTGAACCGGTCTCT
miR-128-qPCR-Reverse	GAGCCATAGTCAAGTTCTCCA
Oligo-dT	TTTTTTTTTTTTTTTTTT
U6-RT	GTCGTATCCAGTGCAGGGTCCGAGGTATTCGCACTGGATACGACAAAATATGGAAC
U6-Forward	TGCGGGTGCTCGCTTCGGCAGC
GSPT1-qPCR-Forward	TATCTTTAGTGGAGACGAGGTT
GSPT1-qPCR-Reverse	CATAATGCCAAGTTCACC
WNT3A-qPCR-Forward	GCAGGAGGGCCCAGCGACGCCGCCG
WNT3A-qPCR-Reverse	CGGCGGCGTCGCTGGGCCCTCCTGC
β-actin-qPCR-Forward	CGTGACATTAAGGAGAAGCTG
β-actin-qPCR-Reverse	CTAGAAGCATTTGCGGTGGAC

**Table 2:** Association of lnc-SNHG16 with the clinicopathological features of patients

Variables	No. cases (48)	Lnc-SNHG16 expression		*p*-value
		Low (n)	High (n)	
**Age**				
<50 years	20	9	11	0.883
≥ 50 years	28	12	16	
**Tumor size**				
≥5 cm	27	8	19	0.025^a^
<5 cm	21	13	8	
**TNM stage**				
I-II	19	13	6	0.005^a^
III-IV	29	8	21	
**Distant metastasis**				
No	17	12	5	0.006^a^
Yes	31	9	22	
**Lymph node metastasis**				
Absent	16	7	9	1.000
Present	32	14	18	
**Histological grade**				
Well	22	11	11	0.561
Moderately/Poorly	26	10	16	

**a**χ^2^ test. P-values in bold print indicate statistically significant differences.

**Table 3:** Association of miR-128 with the clinicopathological features of patients

Variables	No. cases (48)	miR-128 expression		*p*-value
		Low (n)	High (n)	
**Age**				
<50 years	20	10	10	0.461
≥ 50 years	28	11	17	
**Tumor size**				
≥5 cm	27	16	11	0.014^a^
<5 cm	21	5	16	
**TNM stage**				
I-II	19	3	16	0.002^a^
III-IV	29	18	11	
**Distant metastasis**				
No	17	4	13	0.037^a^
Yes	31	17	14	
**Lymph node metastasis**				
Absent	16	8	8	0.537
Present	32	13	19	
**Histological grade**				
Well	22	8	14	0.343
Moderately/Poorly	26	13	13	

**a**χ^2^ test. P-values in bold print indicate statistically significant differences.

## Abbreviations

miRNA: microRNA
lncRNA: long noncoding RNA
EMT: epithelial-mesenchymal transition
CC: cervical cancer
EdU: 5-ethynyl-2'-deoxyuridine
SNHG16: small nucleolar RNA host gene 16
GSPT1: G1 to S phase transition 1
WNT3A: Wnt family member 3A
CCK8: Cell Counting Kit-8
